# Quantification of aortic stenosis diagnostic parameters: comparison of fast 3 direction and 1 direction phase contrast CMR and transthoracic echocardiography

**DOI:** 10.1186/s12968-017-0339-5

**Published:** 2017-03-07

**Authors:** Juliana Serafim da Silveira, Matthew Smyke, Adam V. Rich, Yingmin Liu, Ning Jin, Debbie Scandling, Jennifer A. Dickerson, Carlos E. Rochitte, Subha V. Raman, Lee C. Potter, Rizwan Ahmad, Orlando P. Simonetti

**Affiliations:** 1 Dorothy M. Davis Heart and Lung Research Institute, The Ohio State University, Columbus, OH USA; 2 InCor Heart Institute, University of São Paulo Medical School, São Paulo, SP Brazil; 3 Department of Electrical and Computer Engineering, The Ohio State University, Columbus, OH USA; 4 Siemens Healthcare, Erlangen, Germany; 5 Department of Internal Medicine, Division of Cardiovascular Medicine, The Ohio State University, Columbus, OH USA; 6 Department of Radiology, The Ohio State University, 460 W. 12th Avenue, room 320, 43210 Columbus, OH USA

**Keywords:** Phase contrast imaging, Multi-directional phase contrast CMR, Bayesian model, Aortic stenosis, Transthoracic echocardiography

## Abstract

**Background:**

Aortic stenosis (AS) is a common valvular disorder, and disease severity is currently assessed by transthoracic echocardiography (TTE). However, TTE results can be inconsistent in some patients, thus other diagnostic modalities such as cardiovascular magnetic resonance (CMR) are demanded. While traditional unidirectional phase-contrast CMR (1Dir PC-CMR) underestimates velocity if the imaging plane is misaligned to the flow direction, multi-directional acquisitions are expected to improve velocity measurement accuracy. Nonetheless, clinical use of multidirectional techniques has been hindered by long acquisition times. Our goal was to quantify flow parameters in patients using 1Dir PC-CMR and a faster multi-directional technique (3Dir PC-CMR), and compare to TTE.

**Methods:**

Twenty-three patients were prospectively assessed with TTE and CMR. Slices above the aortic valve were acquired for both PC-CMR techniques and cine SSFP images were acquired to quantify left ventricular stroke volume. 3Dir PC-CMR implementation included a variable density sampling pattern with acceleration rate of 8 and a reconstruction method called ReVEAL, to significantly accelerate acquisition. 3Dir PC-CMR reconstruction was performed offline and ReVEAL-based image recovery was performed on the three (x, y, z) encoding pairs. 1Dir PC-CMR was acquired with GRAPPA acceleration rate of 2 and reconstructed online. CMR derived flow parameters and aortic valve area estimates were compared to TTE.

**Results:**

ReVEAL based 3Dir PC-CMR derived parameters correlated better with TTE than 1Dir PC-CMR. Correlations ranged from 0.61 to 0.81 between TTE and 1Dir PC-CMR and from 0.61 to 0.87 between TTE and 3Dir-PC-CMR. The correlation coefficients between TTE, 1Dir and 3Dir PC-CMR V_peak_were 0.81 and 0.87, respectively. In comparison to ReVEAL, TTE slightly underestimates peak velocities, which is not surprising as TTE is only sensitive to flow that is parallel to the acoustic beam.

**Conclusions:**

By exploiting structure unique to PC-CMR, ReVEAL enables multi-directional flow imaging in clinically feasible acquisition times. Results support the hypothesis that ReVEAL-based 3Dir PC-CMR provides better estimation of hemodynamic parameters in AS patients in comparison to 1Dir PC-CMR. While TTE can accurately measure velocity parallel to the acoustic beam, it is not sensitive to the other directions of flow. Therefore, multi-directional flow imaging, which encodes all three components of the velocity vector, can potentially outperform TTE in patients with eccentric or multiple jets.

**Electronic supplementary material:**

The online version of this article (doi:10.1186/s12968-017-0339-5) contains supplementary material, which is available to authorized users.

## Background

In calcific or degenerative aortic stenosis (AS), the valve undergoes an inflammation process, which culminates with progressive leaflet calcification and reduced excursion, causing a narrowing of the valvular opening. AS has become one of the most frequent cardiac valvular heart diseases in developed countries, and its prevalence is expected to increase due to aging of the population [1]. Accurate quantification of aortic valve stenosis and assessment of clinical symptoms is crucial in making management decisions since untreated severe and/or symptomatic stenosis is related to poor prognosis and low survival rates over 5 years [2].

Clinical grading of AS is currently performed non-invasively by Doppler Transthoracic Echocardiography (TTE) through measurement of aortic peak velocity (V_peak_), mean transaortic pressure gradient (MG), and effective aortic valve area (AVA) [3]. V_peak_ is measured using continuous wave Doppler in multiple acoustic windows, in the search for the perfect alignment of the acoustic beam parallel to the stenotic jet. Gradients are calculated from the peak velocity profile to estimate the pressure difference between the left ventricle and the aorta. Peak gradient (PG) is derived from the highest measured systolic velocity, while MG time-averages the peak gradient over the systolic ejection period. Finally, AVA calculations are performed based on the principle of conservation of mass using the continuity equation, which considers that fluid passing through the left ventricle outflow tract (LVOT) must be equal to fluid crossing the aortic valve. TTE is the clinical modality of choice for AS severity assessment, and the echocardiographic parameters have been validated in comparison to invasive data and proven to be predictors of clinical outcome [4]. However, TTE has been shown to be suboptimal in up to 30% of patients [5] primarily due to limited acoustic windows. In the setting of aortic stenosis, loss of accuracy can be explained not only by poor acoustic windows, but also by misalignments between the ultrasound beam and flow direction, as well as incorrect estimation of the LVOT area used for AVA calculation based on the continuity equation.

**Fig. 1:**
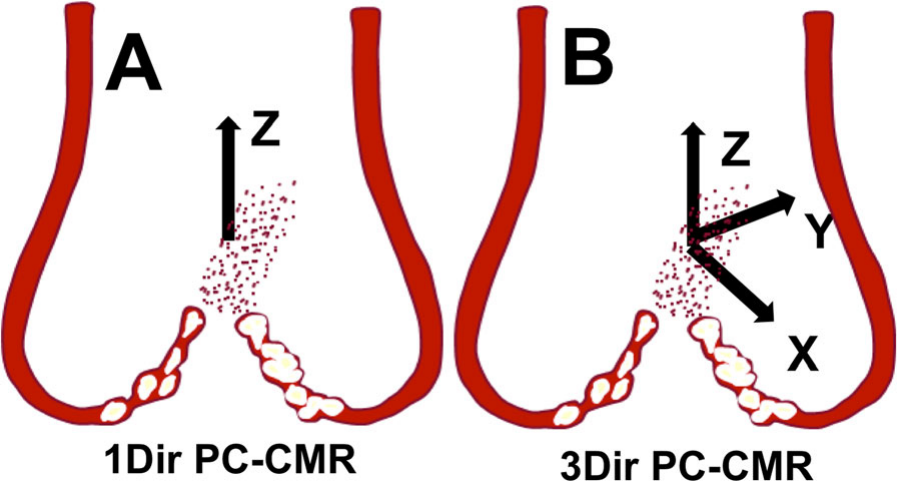
Illustration of the advantage of 3Dir over 1Dir PC-CMR. While 1Dir PC-CMR only computes velocity in one direction (Z), 3Dir PC-CMR simultaneously computes velocities in 3 directions (X, Y, and Z)

Cardiovascular magnetic resonance (CMR) has recently emerged as an important diagnostic modality for noninvasive evaluation of a variety of diseases, including AS [6]. CMR has unique advantages in comparison to TTE, since the entire heart can be visualized without limitations of acoustic windows, and imaging planes can be prescribed in any direction. Flow analysis by CMR typically utilizes an ECG-triggered, segmented k-space, spoiled gradient-echo phase-contrast CMR technique (1Dir PC-CMR) only capable of quantifying velocities in a single direction either parallel or perpendicular to a 2D imaging plane (Fig. 1a). 1Dir PC-CMR requires flow to be interrogated exactly perpendicular to the AS jet direction, otherwise V_peak_ is underestimated. Selection of the proper slice orientation can be challenging as the jet direction may vary with respect to the valve orifice. Thus, accurate prescription of flow acquisition is dependent on the correct operator visualization of the stenotic jet and can be challenging in valvular abnormalities associated with multiple or eccentric jets [7, 8]. Additionally, the jet direction may vary throughout the cardiac cycle, requiring compromises in accurate slice orientation. Although in-plane velocity mapping can help guide the correct slice plane prescription, and the operator should be trained to align the velocity encoding with the direction of the jet at end-systole, acquisition of extra datasets is time consuming and positioning may not be accurate. Indeed, flow analysis by 1Dir PC-CMR has already been shown to underestimate velocity measurements by up to 10% on average in comparison to TTE [9–11]. In this context, a rapid PC technique capable of multi-directional velocity quantification (Fig. 1b) would likely improve the accuracy of peak and mean velocity quantification and allow for more accurate estimation of aortic valve stenosis severity. Multi-directional velocity encoding would reduce operator dependency, would be robust to misalignments between imaging planes and flow jets, and would even be more resistant to the flow jet tilting dynamically during the cardiac cycle. Nonetheless, until recently, multi-directional acquisition has been precluded by long scan times, limiting its clinical implementation.

Previous studies have proposed the use of a two-dimensional three-directional phase contrast technique to assess aortic velocities in AS patients [7, 12]. In one study, the long scan time needed for multi-directional encoding hindered breath-hold imaging so multiple signal averages were acquired to reduce respiratory motion artifacts [7]. Parallel imaging permitted faster data acquisition in a single breath-hold in another study; however, a very long 19 heart-beat acquisition was still required while sacrificing spatial resolution [12]. Volumetric multi-directional imaging (4D flow) has also been proposed and would offer the additional advantage of expanded spatial coverage [13, 14] and has been demonstrated in the assessment of valve disease [15]. However, 4D flow currently suffers from long scan times and requires extensive post-processing, making it currently impractical for routine clinical application [16].

We recently described a data sampling strategy called VISTA [17] and an image reconstruction and processing method called ReVEAL [18] to exploit spatiotemporal sparsity and leverage the relationship between encoded and compensated images to enable highly accelerated PC-CMR. The ReVEAL technique (ReVEAL 3Dir PC-CMR) has been previously shown to achieve an 8 to 10 fold acceleration rate for 1Dir PC-CMR, reducing the acquisition time to a short breath-hold of 3 to 4 s. In the present work, we leverage the acceleration provided by the combination of VISTA and ReVEAL to enable 3Dir PC-CMR during a single breath-hold of less than 14 s.

The purpose of this study is to determine whether this faster technique capable of capturing 3 directions of velocity in a 2D image plane in a single breath-hold (3Dir PC-CMR) provides more accurate measurement of aortic velocity, in the setting of aortic stenosis, as compared with the traditional 1Dir PC-CMR, using TTE as the reference standard.

## Methods

All patients 18 years old and older presenting for transthoracic echocardiographic evaluation in our institution were screened for eligibility from February 2014 to August 2015. Echocardiographic exams were performed as part of the routine care at the clinical echocardiographic laboratories. Inclusion criteria were age and a TTE positive for aortic valve calcification or any degree of aortic valve stenosis. Exclusion criteria encompassed uncontrolled atrial fibrillation, current pregnancy, poor echocardiographic image quality, claustrophobia and presence of pacemaker. Patients presenting with reduced ejection fraction (EF) were not excluded from the study since low flow/low gradient AS would similarly impact disease severity classification by either CMR or TTE. Patients meeting enrollment criteria were recruited for a research CMR within 3 months of their clinical TTE exam. Since aortic stenosis is expected to progress slowly (0.3 m/s or 0.1 mm^2^ per year) [19, 20], no significant difference between TTE and CMR measures was expected due to progression of disease over this time. The local ethics committee approved this study, and a written informed consent was obtained from all participants.

### TTE acquisition

All TTE exams were performed in the clinical echocardiography lab by experienced sonographers that hold certification from the American Registry for Diagnostic Medical Sonography according to standard lab protocol that follows guidelines set forth by the American Society of Echocardiography [3], using three different vendor machines (Philips, General Electric, and Siemens). Aortic velocity profiles were interrogated using continuous wave Doppler, and left ventricular outflow tract velocities were interrogated with pulsed wave Doppler. Aortic velocities profiles were acquired from different echocardiographic windows including the apical 3 chamber, apical 5 chamber, suprasternal notch as well as right parasternal view. In addition, the continuous wave Doppler non-imaging Pedoff probe was used to quantify the highest velocity. The envelope with the highest velocity was used for quantification of peak aortic velocities, peak and mean aortic gradients, velocity time integrals and aortic valve area using the continuity equation. Peak and mean transvalvular pressure gradients were calculated using the modified Bernoulli equation (ΔP = 4 V^2^), where ΔP is pressure gradient and V is peak velocity. Mean gradient was calculated by integrating the equation over time. LVOT area (A_LVOT_) was estimated by measuring the LVOT diameter, D, on a parasternal long-axis view according to ${\mathrm{A}}_{\mathrm{LVOT}} = \pi \cdot {\left(\frac{D}{2}\right)}^2$, assuming a circular LVOT shape. Then, aortic valve area was estimated by the continuity equation AVA = A_LVOT *_ VTI_LVOT_/VTI_AV_, where VTI is the velocity time integral at the LVOT (VTI_LVOT_) and aortic valve (VTI_AV_) levels. Echocardiographic data analysis was performed by the sonographer at the time of the clinical TTE, and clinically reported valve hemodynamic measurements were utilized as the reference standard for comparison with CMR.

### CMR acquisition

CMR was performed using a 1.5-Tesla CMR scanner (Avanto, Siemens Healthineers; Erlangen, Germany) and a 12-channel phased-array coil. Steady state free precession (SSFP) cine images were acquired in two orthogonal planes (3-chamber and LVOT views) for localization of aortic valve and visualization of systolic jets. Additionally, short-axis cine images covering the left ventricular cavity were acquired for stroke volume (SV) calculation using Simpson’s Method.

**Fig. 2:**
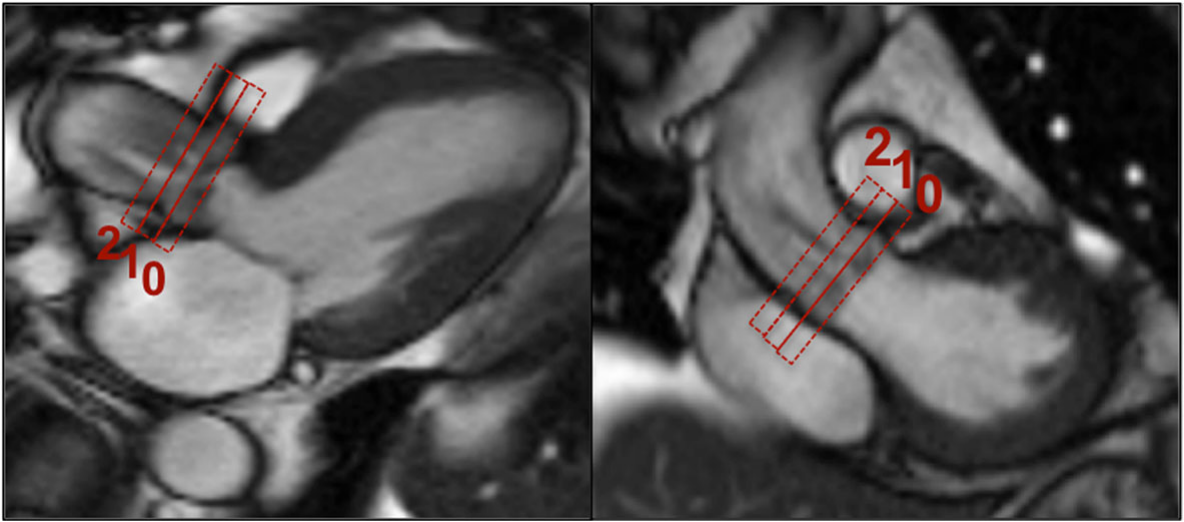
Flow acquisition planes *(rectangles*) are depicted for both PC-CMR techniques. Note the presence of two aortic jets secondary to complex valve geometry. (see also Additional file 1)

1Dir PC-CMR and 3Dir PC-CMR data were acquired at three contiguous levels (0,1,2) just above the aortic valve, with acquisition planes oriented perpendicular to the aortic root anatomy (Fig. 2). Acquisition parameters are listed in Table 1. The center of the first acquisition plane (plane 0) was placed perpendicular to the tips of the aortic valve, using the perpendicular end-systolic three-chamber and LVOT cine images as a guide. The second and third planes were positioned just above plane 0, with no gap. Also a fourth plane was positioned just below the aortic annulus, at the level of the LVOT. A velocity encoding (V_enc_) scout was first acquired using V_enc_ of 200, 300 and 400 cm/s to optimize the V_enc_ setting, followed by 1Dir PC-CMR acquisition. If velocity aliasing was detected on any 1Dir PC-CMR acquisition plane, additional flow images were acquired after increasing V_enc_ until no aliasing was observed. Subsequently, the same optimized V_enc_ was also applied to 3Dir PC-CMR acquisition at the same acquisition plane. The V_enc_ was set the same in all three encoded directions for 3Dir PC CMR in order to keep the echo time short as possible. V_enc_ ranged from 200 to 500 cm/s. 1Dir PC-CMR and 3Dir PC-CMR were collected in separate breath-holds. Balanced four-point encoding with prospectively undersampled VISTA sampling (*R* = 8) was used to collect data for 3Dir PC-CMR. Spatial resolution was matched between PC-CMR techniques. 1Dir PC-CMR was reconstructed online on the scanner using GRAPPA (*R* = 2), while 3Dir PC-CMR raw data was saved and reconstructed offline using Matlab (The Mathworks, Natick, MA, USA).

**Table 1:** Imaging Parameters

Parameter	1Dir PC-CMR	3Dir PC-CMR
Temporal Resolution (ms)	52.25	37.12
TE (ms)	2.3	2.77
TR	5.23	4.64
Lines per segment	5	2
Flip Angle	25°	15°
Echo asymmetry	33% before echo	33% before echo
Bandwidth (Hz/pixel)	420	558
Venc (cm/s)	150–500	150–500
Slice Thickness (mm)	8.0	8.0
Triggering	Retrospective	Prospective
Matrix	144 × 192	128 × 160
FOV (mm)	284 × 374	250 × 313
Pixel dimensions (mm x mm)	1.97 × 1.95	1.95 × 1.96
Acceleration factor	GRAPPA R = 2	VISTA R = 8
Average scan time	17 s	10s

*TE* Echo time, *TR* repetition time, *Venc* Velocity encoding, *FOV* Field of view (phase x frequency encode directions)

### ReVEAL based 3Dir PC-CMR reconstruction

The undersampled k-space data were copied from the scanner and processed offline in Matlab. The data from each velocity encoding direction were paired with the velocity compensated data and processed using ReVEAL, which not only exploits the spatiotemporal structure in the image but also utilizes the redundancies between the encoded and the compensated images. The reconstruction process was repeated for three orthogonal encoding directions. The tuning parameters for ReVEAL, including the regularization strength, were adjusted using data acquired in one healthy volunteer (not shown) and were kept constant for all datasets included in this work. The reconstruction time for a 3Dir PC-CMR was 10 to 15 min/plane using CPU-based processing.

### CMR post-processing

Valve contours were manually traced using the freely available software Segment version 2.0 R4494 (*http://segment.heiberg.se*) [21], and quantitative image analysis was performed using Matlab. Valve segmentation and flow data generation took up to 5 min per dataset. 3Dir PC-CMR peak velocity was calculated pixel by pixel using the equation V = √V_x_^2^ + V_y_^2^ + V_z_^2^. It is known that the peak velocity is susceptible to noise, and presence of even a single noisy pixel can compromise its accuracy. To eliminate pixels that resided outside the blood pool or were obviously corrupted by noise, minimum thresholds were set for total phase accumulation (temporal average of the phase) and magnitude. Reasonable magnitude and flow thresholds were empirically learned from one of the datasets and then uniformly applied to all datasets. After thresholding, the pixel presenting the greatest maximum velocity in each frame was selected to plot the peak velocity (V_peak_) curve. The plane yielding the highest V_peak_ was selected for comparison with TTE. Mean velocity (V_mean_) was calculated similar to the method used in echocardiography, by first finding the peak velocity in each temporal frame, and then averaging across the cardiac cycle. Peak and mean gradients were calculated using the modified Bernoulli equation similarly to TTE. Velocity time integrals (VTIs) were calculated by integrating aortic peak velocity curves over time. No correction of background phase offset was applied; however, a phase unwrapping algorithm was used to salvage datasets with obvious velocity aliasing. AVA estimations by CMR were performed based on two different approaches already presented elsewhere: AVA_Cine_ and AVA_Flow_, both using VTI data from the aortic plane presenting the highest V_peak_ on PC imaging [10]. AVA_Cine_ uses SV data calculated by cine imaging (AVA_Cine_ = Cine SV/PC VTI_AV_), while AVA_Flow_ uses SV quantification by PC-CMR at the same aortic level where the highest V_peak_ was present (AVA_Flow_ = PC SV/PC VTI_AV_).

Statistical analyses were performed using MedCalc 14.8.1 (MedCalc Software, Ostend, Belgium). Variables were tested for normal distribution using the Kolmogorov-Smirnov Test. Linear regression was used for comparison between CMR and TTE measurements, and the Pearson correlation coefficient was reported. Additionally, agreement between CMR techniques and TTE was tested by Bland-Altman analysis and biases ± standard deviations were determined.

## Results

A total of 23 patients (13 men, 10 women, median age 68y) were included in the study. The average time elapsed between TTE and CMR was 36 days (range: 0 to 86 days). The patient population exhibited a good distribution in terms of severity of aortic stenosis, even after exclusions, with 12 (52%) patients classified by TTE as having moderate or severe disease and the remaining having mild stenosis or calcific degenerative valve disease, but no stenosis. Additionally, 52% of patients also presented echocardiographic evidence of mild or moderate aortic regurgitation. Patient characteristics can be found on Table 2. Cardiovascular comorbidities were common, with hypertension affecting 91% and hyperlipidemia 83% of patients. Among all subjects, 35% presented symptoms thought to be related to valvular disease. Of note, controlled atrial fibrillation was present in 4 subjects and did not preclude CMR image acquisition. Data from two patients were not included in the analysis, one presenting mild and another presenting severe AS, due to severe aliasing in at least one 3Dir PC-CMR acquisition plane. Also, three more patients were excluded from specific parameter sub-analyses, one from SV derived parameter analyses due to lack of short-axis cine imaging and two from AVA analyses respectively due to significant sub-valvar velocity acceleration and LVOT diameter overestimation, leading to incorrect AVA estimations by TTE.

**Table 2:** Patient Characteristics

Total number of patients	23 patients
Median age in years (range)	68 (27–85)
Gender – male, n (%)	13 (56%)
LVEF, % (TTE)	59 (37–71%)
LVEF ≤ 50%, n (%)	2 (9%)
HTN, n (%)	21 (91%)
Diabetes, n (%)	5 (22%)
Hyperlipidemia, n (%)	19 (83%)
Documented CAD	9 (39%)
Controlled atrial Fibrillation, n (%)	4 (17%)
AS related symptoms	8 (35%)
Valve Morphology	
Tricuspid, n (%)	19 (83%)
Bicuspid, n (%)	4 (17%)
Aortic Stenosis severity (TTE)	
No stenosis	3 (13%)
Mild, n (%)	7 (30%)^a^
Moderate, n (%)	9 (39%)
Severe, n (%)	4 (17%)^a^
Aortic Regurgitation (TTE)	
No regurgitation	11 (48%)
Mild, n (%)	12 (52%)

*LVEF* Left ventricular ejection fraction, *HTN* Systemic hypertension, *CAD* Coronary artery disease

^a^Two cases excluded from further analysis due to severe aliasing precluding successful phase unwrapping, one mild and one severe stenosis case

**Fig. 3:**
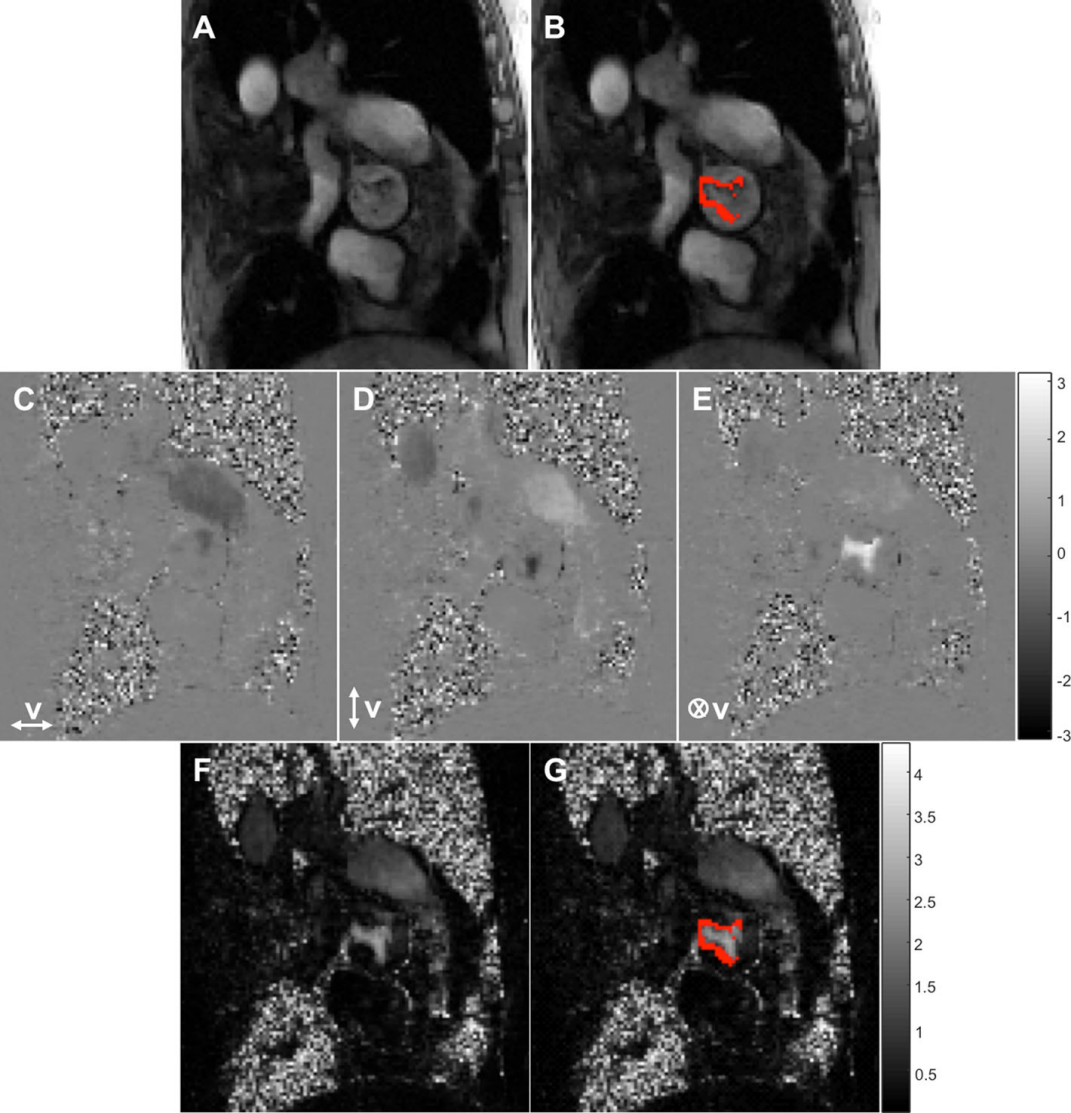
Representative 3Dir PC-CMR images in a patient with mild aortic stenosis (V_peak_ = 2.75 m/s) using ReVEAL-based image recovery (see also Additional file 2). (**a**) The minimum magnitude image obtained by taking the pixel-wise minima across the magnitude images from different encodings, (**b**) the image in (**a**) with the thresholded pixels highlighted in red, (**c**, **d**, **e**) phase images in three encoding directions, Vx, Vy, Vz (**f**) the speed map and (**g**) the image in (**f**) with the thresholded pixels highlighted in red. The discarded pixels have either small magnitude (for one or more velocity components) or insignificant flow

Example magnitude, phase and speed 3Dir PC-CMR images in a patient with mild aortic stenosis are shown in Fig. 3 and Additional file 2.

Kolmogorov-Smirnov Goodness-of-Fit Test results showed normality of data distribution for all variables.

Although good correlations were observed between TTE and both 1Dir PC-CMR and ReVEAL based 3Dir-PC-CMR derived parameters, a significant improvement in all correlations was observed for 3Dir PC-CMR. Pearson’s coefficients ranged from 0.61 to 0.81 for 1Dir PC-CMR and from 0.61 to 0.87 for 3DirPC-CMR. Table 3 summarizes the comparison of CMR techniques and TTE, with Pearson Correlation coefficients (r) and means + − standard deviations provided.

**Table 3:** Comparison of 1Dir and 3Dir PC-CMR derived parameters with TTE

	1Dir PC-CMR x TTE				3Dir PC-CMR x TTE				Comparisons of r
	*r*	95% CI	*p*-value	Bias ± SD	*r*	95% CI	*p*-value	Bias ± SD	*p*-value
Vmean	0.77	0.50–0.90	<0.0001	−0.5 ± 0.4 m/s	0.80	0.56–0.91	<0.0001	−0.2 ± 0.4 m/s	0.6541
Vpeak	0.81	0.58–0.92	<0.0001	−0.2 ± 0.5 m/s	0.87	0.71–0.95	<0.0001	0.2 ± 0.4 m/s	0.5117
MG	0.79	0.55–0.91	<0.0001	−9.5 ± 9.3 mmHg	0.83	0.62–0.93	<0.0001	−2.9 ± 7.6 mmHg	0.7555
PG	0.78	0.53–0.91	<0.0001	−5.5 ± 13.3 mmHg	0.87	0.69–0.94	<0.0001	4.1 ± 11.2 mmHg	0.4270
VTI	0.72	0.41–0.88	0.0003	−3.9 ± 16.3 cm	0.80	0.56–0.91	<0.0001	1.6 ± 14.6 cm	0.5631
SV^a^	0.75	0.47–0.90	0.0001	9.7 mL ± 17.8 mL	0.81	0.57–0.92	<0.0001	−7. 4 mL ± 13.3 ml	0.6749
AVA_Cine_	0.61	0.22–0.83	0.0056	0.31 ± 0.37 cm^2^	0.61	0.21–0.83	0.0057	0.22 ± 0.33 cm^2^	0.9939
AVA_Flow_	0.64	0.27–0.85	0.0030	0.43 ± 0.32 cm^2^	0.66	0.29–0.86	0.0023	0.09 ± 0.30 cm^2^	0.9427

*r* Pearson’s correlation coefficient; 95% confidence intervaI for r, *SD* standard deviation, *Vmean* Mean velocity, *Vpeak* peak velocity, *MG* Mean Gradient, *PG* peak gradient, *VTI* velocity time integral, *SV* stroke volume, AVA_Cine_ = SV cine/PC-CMR VTI AV, AVA_Flow_ = SV_PC_/PC-CMR VTI AV

^a^SV correlation was compared to Cine SV

V_peak_ was higher in planes 0 and 1 than in plane 2 for both 1Dir and 3Dir PC-CMR techniques. Plane 0 presented highest V_peak_ in 24% of cases for 1Dir PC-CMR and 29% of cases for 3Dir PC-CMR while plane 1 presented highest V_peak_ in 57% of cases for 1Dir PC-CMR and 62% of cases for 3Dir PC-CMR. Discrepancies in planes presenting highest V_peak_ between the 1Dir and 3Dir techniques were found in 57% of cases; this may be explained by slight differences in the depth of expiration as well as slight physiological variations between heartbeats during acquisition.

**Fig. 4:**
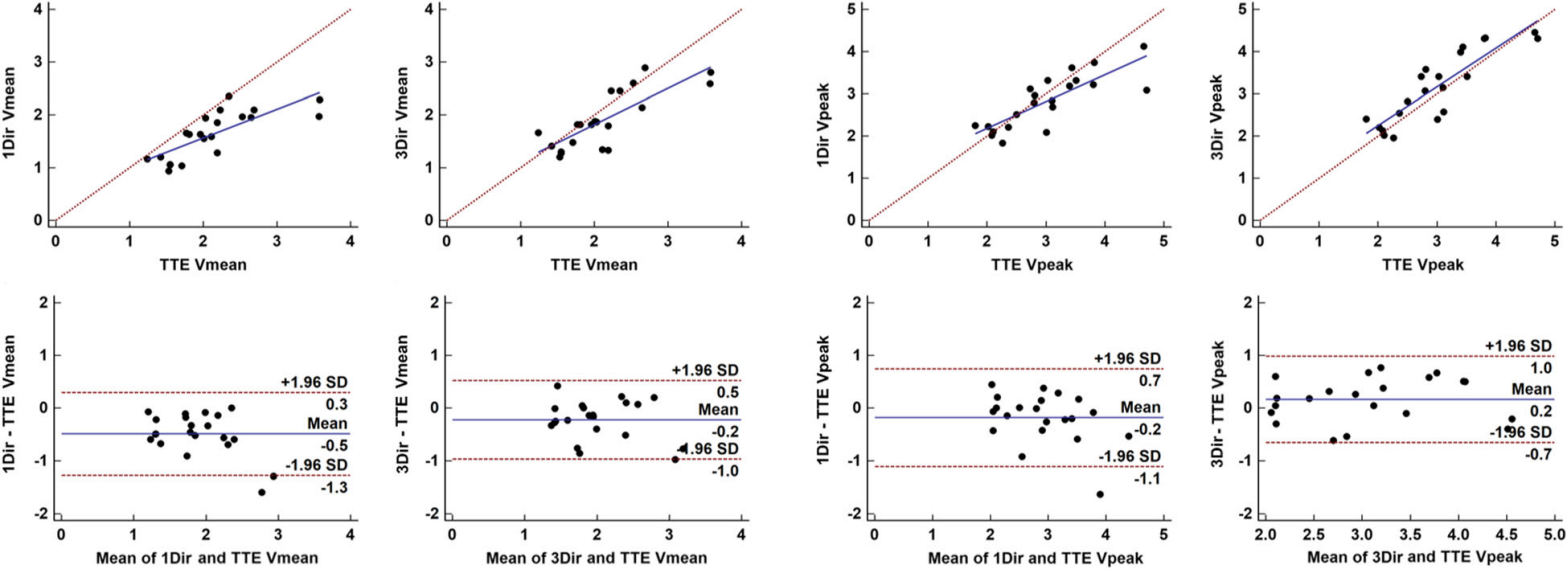
Scatter and Bland-Altman plots of comparison between 1Dir PC-CMR and ReVEAL based 3Dir PC-CMR derived mean and peak velocities versus TTE. Note the underestimation of velocities in moderate-severe cases, with the exception of 3Dir PC-CMR peak velocities

**Fig. 5:**
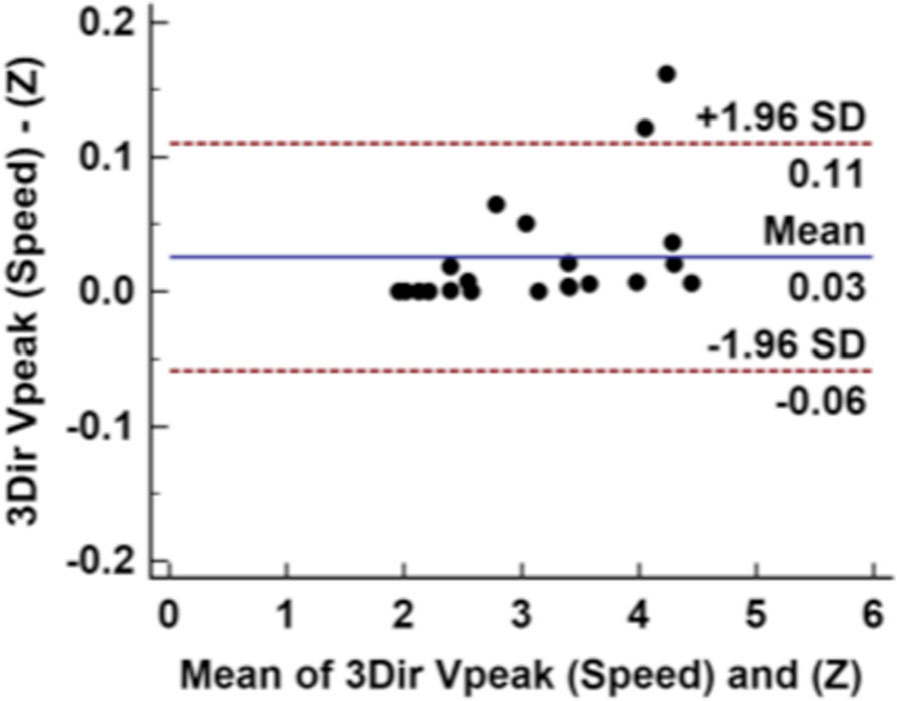
Bland-Altman plot of comparison between 3Dir PC-CMR peak velocities derived from all three velocity components (3Dir PC-CMR speed) and the through-plane component (Z). Differences arise primarily from the 1/3 of the cases where speed was slightly higher than the unidirectional computed peak velocity

V_peak_ was highly correlated with TTE for both 1Dir PC-CMR (*r* = 0.81) and 3Dir PC-CMR (r = 0.87); 1Dir PC-CMR tended to underestimate V_peak_ while 3Dir PC-CMR measured a higher V_peak_ than TTE. Average V_peak_ was 2.8 m/s for 1Dir PC-CMR, 3.17 m/s for 3Dir PC-CMR, and 3.0 m/s for TTE, with a mean difference of −0.18 m/s between 1Dir PC-CMR and TTE, and +0.17 m/s between 3Dir PC-CMR and TTE (Table 3 and Fig. 4). A subanalysis was performed comparing Vpeaks derived from the through-plane 3Dir PC-CMR (Vz direction) only with the vector sum of all velocity components (Fig. 5), to investigate the impact of misalignment on unidirectional velocity estimation using 3Dir Vz as an internal control. We found a mean difference of 0.03 m/s between speed and Vz. This mean difference, although small, reached statistical significance (*p* = 0.0139). The Bland-Altman plot in Fig. 5 demonstrates that the difference was non-zero in about 1/3 of the cases.

V_mean_ correlation with TTE was also higher for 3Dir PC-CMR, although a small negative bias was present for both techniques, with *r* = 0.77 and bias of −0.50 m/s for 1Dir PC-CMR and *r* = 0.80 and bias of −0.23 m/s for 3Dir PC-CMR. Scatter diagrams and Bland-Altman plots in Fig. 4 show that underestimations of velocities occurred in more severe cases where mean and peak velocities were higher, with a clear separation of the trend line from the equality line and increased scatter on the bland-Altman plot with higher velocities. A notable exception for this rule was 3Dir PC-CMR V_peak_, which maintained good agreement, even in more severe cases.

**Fig. 6:**
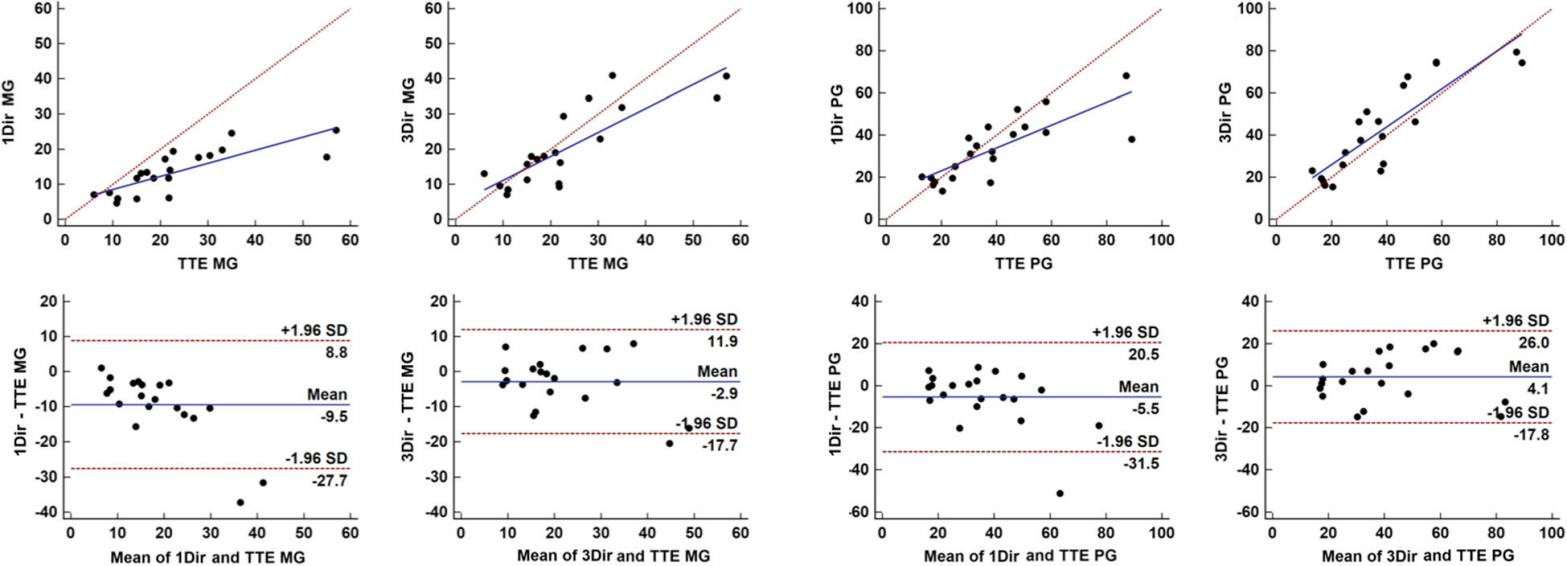
Scatter and Bland-Altman plots of comparison between 1Dir PC-CMR and ReVEAL based 3Dir PC-CMR derived mean and peak gradients versus TTE. The same trend of results was observed for mean and peak gradients when compared to mean and peak velocities

Similar results were observed for mean and peak gradients. MG correlations increased from 0.79 to 0.83 from 1Dir PC-CMR to 3Dir PC-CMR versus TTE and from 0.78 to 0.87 for PG (Table 3). Again, a negative bias with a significant separation of the trend line from the equality line was observed for more severe cases, with a more significant negative bias of -10 mmHg for 1Dir PC-CMR MG in comparison to TTE (Fig. 6). However, 3Dir PC-CMR results maintained a mean difference near zero and narrower limits of agreement for both mean and peak gradients, as depicted on the Bland-Altman plots in Fig. 6.

**Fig. 7:**
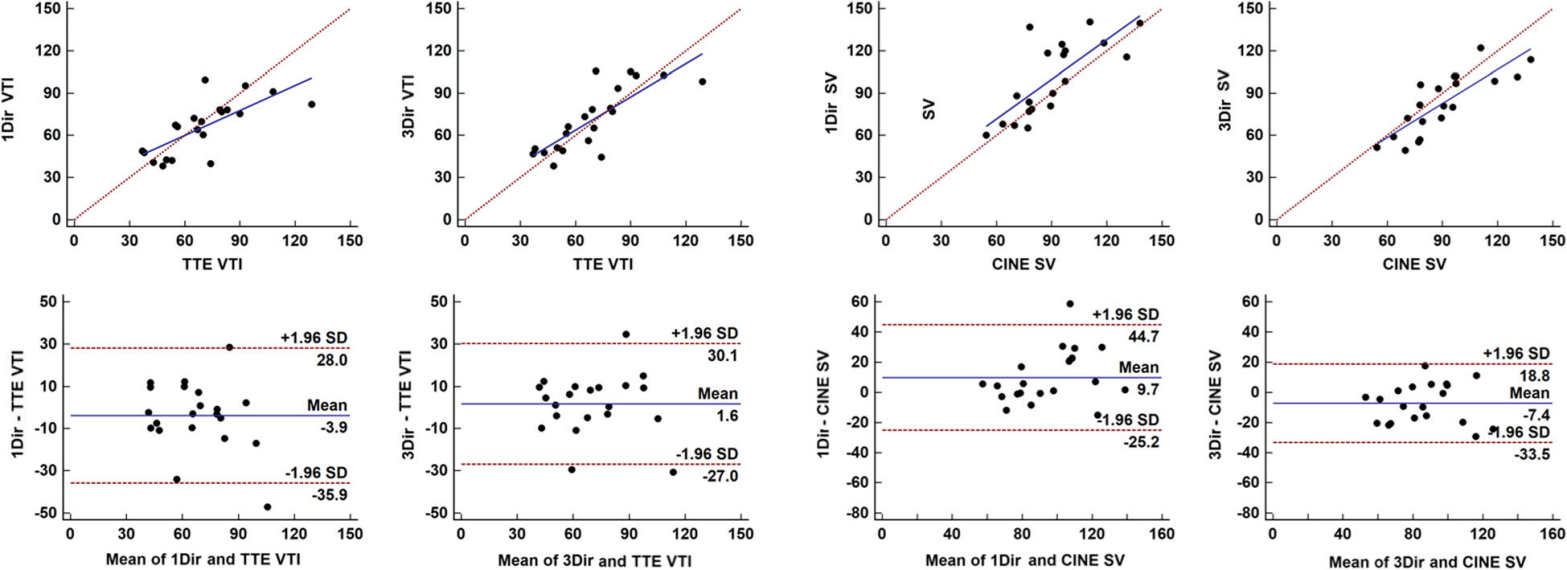
Scatter and Bland-Altman plots of comparison between 1Dir PC-CMR and ReVEAL based 3Dir PC-CMR derived VTI versus TTE and SV results versus SSFP cine imaging

CMR VTI data correlated well with TTE derived VTI (Table 3 and Fig. 7). Although correlation was superior for 3Dir PC-CMR (*r* = 0.80) in comparison to 1Dir PC-CMR (*r* = 0.72), the limits of agreement were still large for both techniques (±16.3 cm for 1Dir PC-CMR and ± 14.6 cm 3Dir PC-CMR). Stroke volumes measured from planes with highest velocities showed a good correspondence with stroke volume measurements by Simpson’s volumetric analysis, although a systematic positive bias was observed for 1Dir PC-CMR (+9.7 ml) and a negative bias was observed for 3Dir PC-CMR (−7.4 ml) (Fig. 7).

**Fig. 8:**
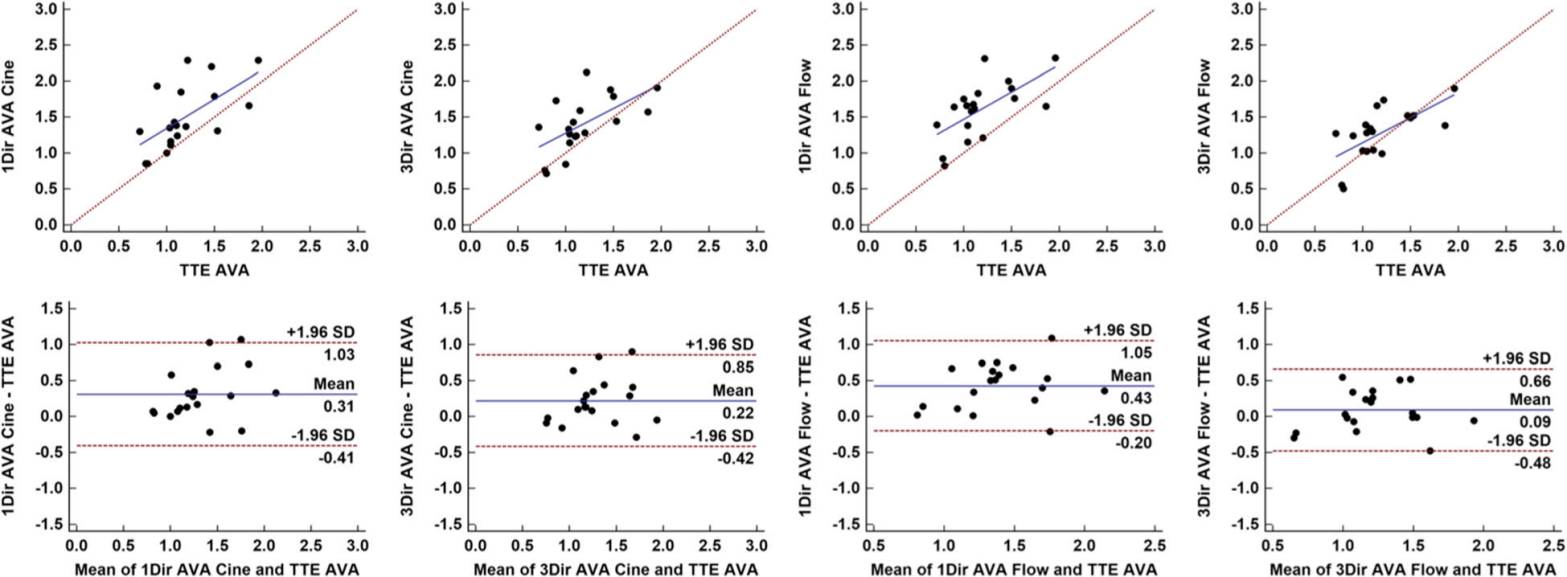
Scatter and Bland-Altman plots of comparison between 1Dir PC-CMR and ReVEAL based 3Dir PC-CMR aortic valve area calculations versus TTE AVA estimates by the continuity equation

Finally, moderate agreement was observed between CMR derived estimates of AVA and TTE, with r ranging from 0.61 to 0.66. However, all CMR methods overestimated AVA in comparison to TTE (Table 3). A positive mean bias was observed for both AVA_Cine_ and AVA_Flow_, which ranged from + 0.09 to + 0.43 cm^2^ (Table 3). Mean bias was the smallest (+0.09 cm^2^) for 3Dir PC-CMR AVA_flow_ quantification (Fig. 8), but at the expense of SV underestimation by this technique.

When a sub-analysis was performed with only moderate and severe cases, 1Dir PC-CMR showed moderate correlations to TTE while 3Dir PC-CMR showed moderate to good correlations, with r ranging from 0.63 to 0.71 for 1Dir PC-CMR and from 0.69 to 0.83 for 3Dir-PC-CMR (Table 4). Although a drop in correlation coefficients was observed for both techniques, it was more pronounced for 1Dir PC-CMR correlations (Tables 3 and 4). Although modest, increase in Pearson’s correlation in this sub-analysis was observed for both 1Dir and 3Dir PC-CMR AVA estimates.

**Table 4:** Sub-analysis in the patient subgroup of moderate and severe aortic stenosis

	1Dir PC-CMR x TTE				3Dir PC-CMR x TTE				Comparisons of r
	*r*	95% CI	*p*-value	Bias ± SD	*r*	95% CI	*p*-value	Bias ± SD	*p*-value
Vmean	0.63	0.09–0.88	0.0276	−0.6 ± 0.5 m/s	0.76	0.33–0.93	0.0040	−0.3 ± 0.4 m/s	0.5852
Vpeak	0.70	0.22–0.91	0.0107	−0.4 ± 0.5 m/s	0.83	0.48–0.95	0.0009	0.2 ± 0.4 m/s	0.5229
MG	0.71	0.23–0.91	0.0093	−13.5 ± 10.5 mmHg	0.79	0.39–0.94	0.0022	−4.6 ± 8.6 mmHg	0.7048
PG	0.68	0.17–0.90	0.0151	−10.2 ± 15.4 mmHg	0.82	0.47–0.95	0.0011	3.9 ± 12.3 mmHg	0.4843
VTI	0.69	0.19–0.91	0.0128	−10.3 ± 16.0 cm	0.76	0.32–0.93	0.0045	−1.3 ± 15.0 cm	0.7726
SV^a^	0.64	0.11–0.89	0.0250	10.8 ± 21.7 ml	0.77	0.34–0.93	0.0037	−10.0 ± 15.7 ml	0.5958
AVA_Cine_	0.70	0.17–0.91	0.0171	0.36 ± 0.39 cm2	0.69	0.16–0.91	0.0184	0.23 ± 0.35 cm2	0.9831
AVA_Flow_	0.68	0.13–0.91	0.0216	0.46 ± 0.35 cm2	0.73	0.24–0.93	0.0099	0.06 ± 0.27 cm2	0.8197

*r* Pearson’s correlation coefficient; 95% confidence interval for r, *SD* standard deviation, *Vmean* Mean velocity, *Vpeak* peak velocity, *MG* Mean Gradient, *PG* peak gradient, *VTI* velocity time integral, *SV* stroke volume. AVA_Cine_ = SV cine/PC-CMR VTI AV, AVA_Flow_ = SV_PC_/PC-CMR VTI AV

^a^SV correlation was compared to Cine SV

In general, better correlations were observed for 3Dir PC-CMR and TTE. Despite that, the comparisons of Pearson’s correlations between the techniques did not reach statistical significance, even in the sub-analysis of more severe cases, with *p*-values > 0.05 (Comparisons of r on Tables 3 and 4).

## Discussion

Overall, good correlations were found between TTE, 1Dir PC-CMR and ReVEAL based 3Dir-PC-CMR parameters, with improvements in correlations observed for most 3Dir PC-CMR parameters.

The higher V_peaks_ measured by 3Dir PC-CMR can be explained by its multidirectional capability. Since 3Dir PC-CMR accounts for velocity in any direction, it may be more accurate than both 1Dir PC-CMR and TTE in the clinical assessment of AVS, since both techniques are sensitive to operator defined orientation of data acquisition. Similar trends in results from V_peak_, MG and PG were observed, likely a result of the methods used to estimate mean and peak gradient by the Bernoulli equation, causing any velocity errors to be squared.

CMR V_mean_ and VTI calculations are inherently different from TTE calculations. While in TTE the velocity signal can come from anywhere along the acoustic beam path, CMR V_mean_ and VTI are derived from the pixels within a 2D planar region of interest.

AVA calculations from AVA_flow_ and AVA_cine_ approaches based on cine and PC-CMR at the aortic valve level are very attractive clinically, because cine imaging and aortic flows are already acquired routinely and do not require acquisition of additional LVOT data. CMR derived LVOT results have been previously shown to be extremely dependent on slice plane location within the LVOT [22]. On TTE, the Doppler sample volume is normally positioned in the LVOT where laminar flow is present, as the sample volume is moved away from the valve towards LV apex. Also, LVOT measures by TTE assume that LVOT has a homogeneous and flat velocity profile, while previous CMR work reveals that LVOT flow is skewed, with higher velocities found closer to the septum and lower velocities closer to mitral valve [23].

When data analysis included only moderate and severe AS cases, the discrepancy in correlations between 1Dir and 3Dir PC-CMR versus TTE were even more evident. This sub-analysis more clearly reflects the everyday clinical dilemma since patients have a higher probability of being referred for additional advanced imaging when TTE results are discrepant between each other and/or with clinical data, while mild cases are generally followed by TTE, a cheaper and more readily available technique. Thus, gains in PC-CMR accuracy in moderate and severe AS cases may actually be more clinically relevant than in mild cases.

Although TTE is the clinical gold standard modality for assessment of AS due to its accuracy, portability, and reasonable cost, TTE has a number of limitations. Doppler interrogation of valve velocity should be performed in a direction parallel to flow, which requires the sonographer to search for the best acquisition window and for the best V_peak_ envelope by manipulating and tilting the transducer on the chest wall [3]. However, poor echocardiographic windows, unfavorable anatomic variations (valvular asymmetric openings, horizontal heart positions, etc.) or lung disease may preclude exact parallel orientation of the Doppler beam with the high-velocity aortic jets. Additionally, TTE frequently cannot directly visualize the stenotic valve opening with sufficient quality. TTE makes assumptions based on the geometric area of the LVOT and approximations with the continuity equation are used. For this reason, the area estimation by the continuity equation is considered to be effective and takes into account flow contraction through the stenotic orifice. When LVOT diameter is squared for calculation of LVOT cross sectional area, it becomes the greatest potential source of error in the continuity equation [3].

CMR, on the other hand, has unique advantages in comparison to TTE, since it does not suffer from unfavorable acquisition windows and can be acquired in any direction [6]. However, CMR is typically used clinically in the subgroup of patients with moderate and severe disease as an alternative to more invasive techniques (cardiac catheterization and transesophageal echo) when TTE results are equivocal. Our data showed that patients with moderate and severe disease may benefit the most from multidirectional acquisition, perhaps because severe jets may be more likely to be oriented in non-orthogonal directions with respect to the valve. Multidirectional velocity encoding makes prescription of the imaging plane less operator dependent [7]. Additionally, single direction encoding cannot accommodate a jet that changes direction across the cardiac cycle; in such cases any slice orientation is a compromise.

It has previously been suggested that 3Dir velocity encoding would be a more rigorous method to measure V_peak_, but the increased acquisition time or severe compromises required in spatial and temporal resolution have prevented practical application. 4D flow has also been proposed as a slice orientation independent technique, but scan times as long as 7 to 15 minutes have been necessary to assess flow in aortic stenosis [13, 14]. The highly accelerated 3Dir PC-CMR cine images produced from the combination of ReVEAL and VISTA allow for multidirectional PC acquisition that is faster than current segmented 1Dir PC-CMR techniques. Importantly, ReVEAL is efficient enough to support 3Dir PC-CMR acquisition with adequate spatial and temporal resolution in a reasonable breath-hold without the need for EPI or other alternative k-space trajectories that can induce phase errors. The biggest current disadvantage of ReVEAL based 3Dir PC-CMR is the time required for iterative reconstruction, making it not ready for immediate and widespread clinical application. This limitation should be overcome in the future through implementation of optimized code on parallel computer hardware.

Our study was performed in a relatively small number of patients and has other limitations. First, TTE exams were not performed by a single observer in a controlled research setting. These were clinical echocardiography studies performed by a group of experienced sonographers who routinely perform clinical TTE studies at our institution, strictly following current guidelines [3]. We believe this scenario better reflects the everyday practice, and in the future the performance of 3Dir PC-CMR should be evaluated in a routine clinical setting and performed by MR technologists. Another potential limitation of the technique is that the Venc setting for the in-plane directions, where velocities are expected to be relatively low, must be a compromise between the optimal dynamic range provided by lower Venc, and the immunity to dephasing errors afforded by higher Venc. In our study we set the in-plane Venc equal to the through plane Venc, anticipating that dephasing due to acceleration and higher order motion terms would be problematic if the TE were extended to achieve lower Venc. Optimal setting of Venc and TE may require additional investigation.

## Conclusions

In conclusion, we have demonstrated in a small cohort of patients with aortic stenosis that 3-directional velocity encoding can be achieved with reasonable spatial resolution, temporal resolution, and scan time. An improvement in flow derived parameter correlations were observed between 3Dir PC-CMR and TTE, when compared to 1Dir PC-CMR. This was expected as 3Dir PC-CMR accounts for velocity in all directions as opposed to TTE and 1Dir PC-CMR, which both measure velocity in only one operated-defined direction. Multi-directional flow imaging might thus outperform TTE, particularly in patients with eccentric or multiple jets.

## Additional files

Additional file 1: Movie showing jets changing direction across the cardiac cycle and corresponding through-plane phase images. (GIF 6246 kb)
Additional file 2: Movie showing 3Dir ReVEAL based PC-CMR magnitude, phase (Vx, Vy, Vz) and speed images in this same mild aortic stenosis case. (GIF 3010 kb)

## Abbreviations

AS: Aortic stenosis
AVA: Aortic valve area
CAD: Coronary artery disease
CMR: Cardiovascular magnetic resonance
EF: Ejection fraction
GRAPPA: Generalized Autocalibrating Partially Parallel Acquisitions
HTN: Hypertension
LVOT: Left ventricular outflow tract
MG: Mean gradient
PC: Phase contrast
PG: Peak gradient
ReVEAL: Reconstructing velocity encoded MRI with Approximate message passing aLgorithms
SSFP: Steady state free precession
SV: Stroke volume
TE: Echo time
TTE: Transthoracic echocardiography
V_enc_: Velocity enconding
VISTA: Variable density incoherent spatiotemporal acquisition
Vmean: Mean velocity
V_peak_: Peak velocity
VTI: Velocity time integral
