# Supplementary figures and images for: Quantification of aortic stenosis diagnostic parameters: comparison of fast 3 direction and 1 direction phase contrast CMR and transthoracic echocardiography
Source: J Cardiovasc Magn Reson. 2017 Mar 7;19:35. doi: 10.1186/s12968-017-0339-5 (PMC5339981; doi:10.1186/s12968-017-0339-5)

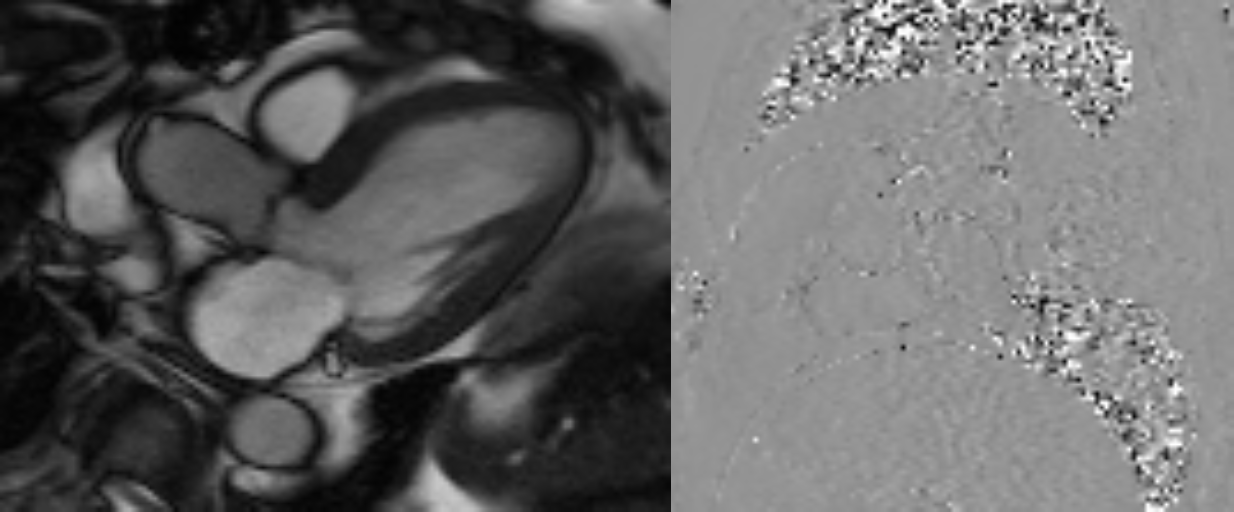

Supplement: Additional file 1: — Movie showing jets changing direction across the cardiac cycle and corresponding through-plane phase images. (GIF 6246 kb) [file 12968_2017_339_MOESM1_ESM.gif]

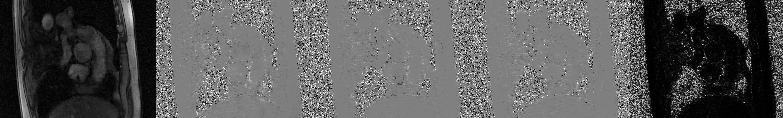

Supplement: Additional file 2: — Movie showing 3Dir ReVEAL based PC-CMR magnitude, phase (Vx, Vy, Vz) and speed images in this same mild aortic stenosis case. (GIF 3010 kb) [file 12968_2017_339_MOESM2_ESM.gif]
